# Heart Rate Fluctuation and Mortality in Critically Ill Myocardial Infarction Patients: A Retrospective Cohort Study

**DOI:** 10.3389/fcvm.2021.577742

**Published:** 2021-05-14

**Authors:** Qi Guo, Hongwei Li, Huijun Ouyang, Runlu Sun, Junjie Wang, Maoxiong Wu, Yue Pan, Jingfeng Wang, Yuling Zhang

**Affiliations:** ^1^Department of Cardiology, Sun Yat-sen Memorial Hospital of Sun Yat-sen University, Guangzhou, China; ^2^Guangdong Provincial Key Laboratory of Arrhythmia and Electrophysiology, Sun Yat-sen Memorial Hospital of Sun Yat-sen University, Guangzhou, China; ^3^Guangdong Provincial Key Laboratory of Malignant Tumor Epigenetics and Gene Regulation, Sun Yat-Sen Memorial Hospital of Sun Yat-Sen University, Guangzhou, China

**Keywords:** myocardial infarction, mortality, heart rate fluctuation, risk factor, intensive care unit

## Abstract

**Background:** Whether heart rate (HR) fluctuation after admission has an impact on the outcomes of critically ill myocardial infarction (MI) patients in intensive care unit remains unknown.

**Methods:** A total of 2,031 MI patients were enrolled from the Medical Information Mart for Intensive Care (MIMIC-III) database. HR fluctuation was calculated as the maximum HR minus the minimum HR in the initial 24 h after admission. Participants were divided into 3 groups, namely, low HR fluctuation [<30 beats per minute (bpm)], medium HR fluctuation (30–49 bpm), and high HR fluctuation (≥ 50 bpm). The main outcomes were 30–day and 1-year mortality. Cox regression and restricted cubic spline model were used.

**Results:** Each 10-bpm increase in HR fluctuation was associated with a higher risk of 30-day mortality and 1-year mortality, with adjusted hazard ratios of 1.122 (95% CI, 1.083–1.162) and 1.107 (95% CI, 1.074–1.140), respectively. Compared with the low HR fluctuation group, the high HR fluctuation group suffered a significantly higher risk of mortality after adjustment, with hazard ratios of 2.156 (95% CI, 1.483–3.134) for 30-day mortality and 1.796 (95% CI, 1.354–2.381) for 1-year mortality. A typical J-type curve was observed in restricted cubic splines for the association between HR fluctuation and 30-day or 1-year mortality of MI patients, with the lowest risk on the HR fluctuation of 30 bpm. Sensitivity analyses emphasized the robustness of our results.

**Conclusions:** This retrospective cohort study revealed an independent positive association between HR fluctuation and 30-day and 1-year mortality in critically ill MI patients, which warrants further investigation.

## Introduction

Myocardial infarction (MI) is common in intensive care unit (ICU), resulting to an enormous cost worldwide (1). It is of great importance to perform MI risk stratification to help patients gain benefit and reduce cost. MI patients in the ICU are exposed to different degrees of artificial light, noise, and various organ supports, including ventilation and medications, which might commonly cause disrupted rhythms of sleep architecture, core body temperature, blood pressure, and heart rate (HR) (2). Besides the ICU environments, severe diseases, and acute stress response might further contribute the fluctuation of vital signs (3). However, whether these fluctuations of vital sings have an impact on the outcomes of critically ill MI patients in the ICU has been poorly investigated.

HR is a widely recognized predictor of cardiovascular diseases and outcomes in numerous cohorts of patients with heart failure or patients at high cardiovascular risk after MI (4, 5). As a common kind of physiological change, HR also shows an obvious fluctuation across 24 h. It has been reported that the fluctuation range of HR was an easily available prognostic predictor for chronic heart failure, which might be correlated with autonomic tone and exercise capacity (6). Recently, we reported that both high and low HR fluctuation were associated with higher mortality in ICU (7). Nevertheless, the association between HR fluctuation and the risk of mortality in MI patients remains unknown. We hypothesized that HR fluctuation might be positively correlated with the risk of mortality in MI patients.

To better clarify the association between HR fluctuation and risk of mortality in MI patients, this retrospective cohort study was designed and conducted using a multivariate Cox hazard ratio regression model and restricted cubic spline model based on the Medical Information Mart for Intensive Care (MIMIC-III) database. In addition, detailed sensitivity analyses were carried out to further evaluate the robustness and reliability of our results under different levels of admission HR and the use of vasopressors, sedatives, or other HR-limiting conditions.

## Materials and Methods

### Data Source

The cohort data were retrieved from the MIMIC-III database. Briefly, MIMIC-III integrated comprehensive and de-identified clinical data of 53,423 distinct ICU stays for 38,597 adult patients in the ICU from 2001 to 2012. The overall information was saved as a relational database, consisting of patient demographics, laboratory tests, discharge summaries, electrocardiographs, imaging examinations, diagnostic information [such as the International Classification of Diseases, Ninth Revision (ICD-9) codes], and in-hospital and out-of-hospital mortality. The use of the MIMIC-III database was approved by the review boards of the Massachusetts Institute of Technology and Beth Israel Deaconess Medical Center (8).

### Study Cohort

The Structure Query Language was used for data extraction (9). Patients admitted to the ICU who were diagnosed with MI were eligible for inclusion. After the MIMIC-III database was screened, a total of 2,031 patients with MI were enrolled in our study. For multiple ICU admissions of one patient, only the data of each patient's first ICU admission were extracted. All characteristics were collected in the initial 24-h documents following admission. The following variables for further statistical analysis were included: [1] basic demographics, including age, sex, and weight; [2] HR, sedative use, ventilation use, vasopressor use, simplified acute physiology score (SAPS), and sequential organ failure assessment (SOFA) score; and [3] comorbidities, including hypertension, congestive heart failure (CHF), atrial fibrillation (AF), liver disease, renal disease, chronic obstructive pulmonary disease (COPD), diabetes, depression, and malignancy. Because more than 20% of patients suffered the missing value of height, height and body mass index were not enrolled in this study to avoid bias resulting from missing value.

### HR Fluctuation and Outcomes

Admission HR was identified as the first record of HR after ICU admission, while HR fluctuation was calculated as the maximum HR minus the minimum HR in the initial 24 h. According to the level of HR fluctuation, participants were divided into 3 groups, namely, low HR fluctuation [<30 beats per minute (bpm)], medium HR fluctuation (30–49 bpm), and high HR fluctuation (≥ 50 bpm). In this study, we regarded 30-day mortality and 1-year mortality as the outcome events, which were also extracted from the MIMIC-III database.

### Statistical Analysis

Normally distributed continuous variables are presented as the mean ± standard deviation, while non-normally distributed data are presented as the median [interquartile range (IQR)]. Differences in continuous variables were tested using the analysis of variance test or rank-sum test as appropriate. Categorical variables were presented as numbers (percentages) and tested by the chi-square test. Linear regression was used to evaluate *P* value for trend of each variable across 3 groups with different HR fluctuation. Multivariable Cox hazard regression models with backward processes were used to investigate the association between HR fluctuation and outcomes. Model 1 was adjusted for age, male sex, and weight. Model 2 was adjusted for model 1 plus SOFA score. Model 3 was adjusted for model 2 plus admission HR. Model 4 was adjusted for model 3 plus sedative use, ventilation use, and vasopressor use. Model 5 was adjusted for model 4 plus CHF, AF, hypertension, and liver disease. HR fluctuation was analyzed as both a continuous variable and a categorical variable in our study, aiming to show the association between HR fluctuation and mortality of MI patients more particular and comprehensive. For continuous variables, the hazard ratio and 95% confidence interval (CI) corresponded to a 10-bpm increase in HR fluctuation. For categorical variables, hazard ratios and 95% CIs in the high and medium HR fluctuation groups were calculated and compared with those in the low HR fluctuation group.

The crude and adjusted models using restricted cubic spline with 5 knots were constructed to flexibly represent the association between the hazards and HR fluctuation as a continuous variable, using a reference level of 30 bpm (10). We considered that HR fluctuation may be influenced by the use of anti-hypertensives, sedatives, and vasopressors. Thus, sensitivity analyses were conducted to determine whether the results persisted in subgroups with different admission HRs, with or without hypertension, AF, vasopressor use, ventilation use, and sedative use.

A two-tailed test was performed, and a *P* < 0.05 was considered statistically significant in our study. SPSS software (version 23.0, IBM, NY, USA) and the R tool (version 3.6.3, R Foundation for Statistical Computing, Vienna, Austria) were used for statistical analysis.

## Results

Among 2,031 critically ill patients with MI, the mean age was 67.9 ± 14.2 years, and 64.0% of them were male. Patients with high HR fluctuation were significantly older in age, had lower weight and had higher admission HR (*P* for trend <0.01). Patients with high HR fluctuation had a higher SAPS score of 20.4 ± 6.2 and a higher SOFA score of 5.0 (IQR, 2.0–8.0) (*P* for trend <0.001). Additionally, this group was more likely to have comorbidities of CHF (*P* for trend <0.05). Compared with the low HR fluctuation group, the high HR fluctuation group suffered a higher 30-day mortality (28.5 vs. 6.5%) and 1-year mortality (40.1 vs. 12.7%) (*P* < 0.001) (Table 1).

**Table 1:** Baseline characteristics of enrolled participants grouped by HR fluctuation.

	**Low**	**Medium**	**High**	***P***	***P* for trend**
	**HR fluctuation (< 30 bpm)**	**HR fluctuation (30–49 bpm)**	**HR fluctuation (≥ 50 bpm)**		
Number	632	704	695		
Age, years	65.7 ± 14.1	67.3 ± 14.8	70.1 ± 13.4	<0.001	<0.001
Male	426 (67.4)	460 (65.3)	413 (59.4)	0.007	0.170
Weight, kg	82.9 ± 19.8	80.8 ± 19.3	79.0 ± 19.9	0.001	0.001
SAPS score	14.4 ± 4.7	16.0 ± 5.7	20.4 ± 6.2	<0.001	<0.001
SOFA score	2.0 (1.0–3.0)	2.0 (1.0–5.0)	5.0 (2.0–8.0)	<0.001	<0.001
Admission HR, bpm	78.2 ± 13.1	84.1 ± 14.8	91.8 ± 21.2	<0.001	<0.001
Sedative	94 (14.9)	200 (28.4)	367 (52.8)	<0.001	0.105
Ventilation	98 (15.5)	234 (33.2)	406 (58.4)	<0.001	0.064
Vasopressor	104 (16.5)	199 (28.3)	369 (53.1)	<0.001	0.129
Hypertension	388 (61.4)	413 (58.7)	366 (52.7)	0.004	0.137
CHF	181 (28.6)	263 (37.4)	334 (48.1)	<0.001	0.036
AF	85 (13.4)	140 (19.9)	285 (41.0)	<0.001	0.189
Liver disease	14 (2.2)	23 (3.3)	43 (6.2)	0.001	0.162
Renal disease	46 (7.3)	58 (8.2)	58 (8.3)	0.736	0.275
COPD	53 (8.4)	67 (9.5)	72 (10.4)	0.470	0.037
Diabetes	171 (27.1)	186 (26.4)	180 (25.9)	0.892	0.061
Depression	34 (5.4)	36 (5.1)	22 (3.2)	0.100	0.253
Malignancy	35 (5.5)	41 (5.8)	52 (7.5)	0.281	0.245
30-day mortality	41 (6.5)	64 (9.1)	198 (28.5)	<0.001	0.264
1-year mortality	80 (12.7)	121 (17.2)	279 (40.1)	<0.001	0.235

*Baseline data were presented by 3 groups, namely, low HR fluctuation (<30 bpm), medium HR fluctuation (30–49 bpm), and high HR fluctuation (≥ 50 bpm). Normally distributed continuous variables are presented as the mean ± standard deviation, while non-normally distributed data are presented as the median (interquartile range). HR, heart rate; bpm, beats per minute; SAPS, simplified acute physiology score; SOFA, sequential organ failure assessment; CHF, congestive heart failure; AF, atrial fibrillation; COPD, chronic obstructive pulmonary disease*.

As a continuous variable, each 10-bpm increase in HR fluctuation was associated with a higher risk of 30-day mortality and 1-year mortality, with hazard ratios of 1.214 (95% CI, 1.179–1.250) and 1.193 (1.164–1.222), respectively. After further strict adjustment in model 5, the association remained significant, with hazard ratios of 1.122 (95% CI, 1.083–1.162) and 1.107 (95% CI, 1.074–1.140), respectively (Table 2).

**Table 2:** Hazard ratio and 95% CI of HR fluctuation (continuous) for mortality.

	**30-day mortality**		**1-year mortality**	
	**Hazard ratio (95% CI)**	***P***	**Hazard ratio (98% CI)**	***P***
Model 1	1.214 (1.179–1.250)	<0.001	1.193 (1.164–1.222)	<0.001
Model 2	1.127 (1.089–1.165)	<0.001	1.113 (1.083–1.145)	<0.001
Model 3	1.117 (1.079–1.156)	<0.001	1.104 (1.073–1.136)	<0.001
Model 4	1.122 (1.083–1.162)	<0.001	1.115 (1.082–1.148)	<0.001
Model 5	1.122 (1.083–1.162)	<0.001	1.107 (1.074–1.140)	<0.001

*The hazard ratio and 95% CI were calculated along with a 10-bpm HR fluctuation increase by the Cox hazard regression model using a backward process. Model 1 adjusted for age, male sex, and weight. Model 2 adjusted for model 1 plus SOFA score. Model 3 adjusted for model 2 plus admission HR. Model 4 adjusted for model 3 plus sedative use, ventilation use, and vasopressor use. Model 5 adjusted for model 4 plus CHF, AF, hypertension, and liver disease. HR, heart rate; SAPS, simplified acute physiology score; SOFA, sequential organ failure assessment; CHF, congestive heart failure; AF, atrial fibrillation; CI, confidence interval*.

**Figure 1:**
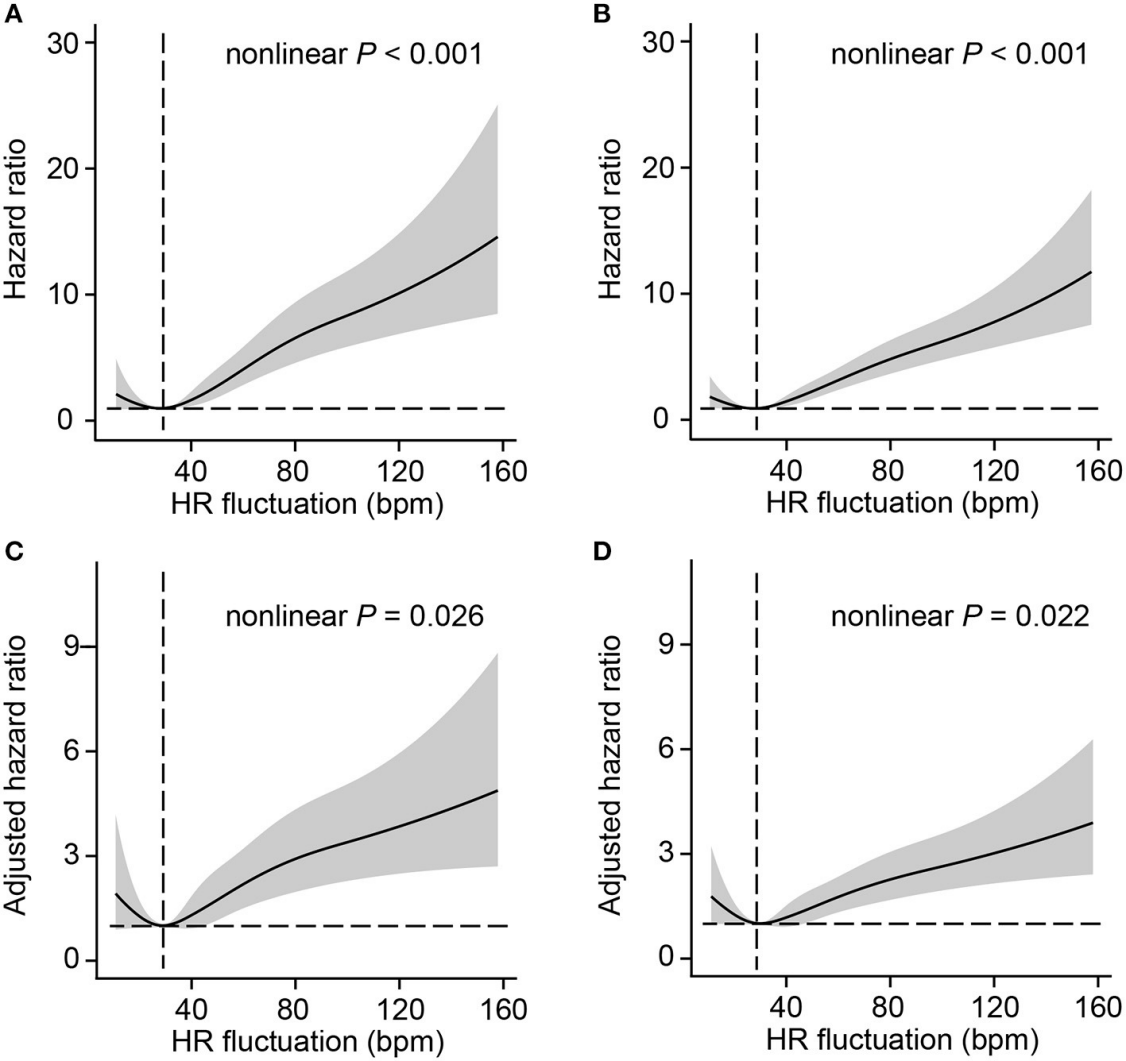
Association between HR fluctuation and outcomes of MI patients. Crude hazard ratio and 95% CI for HR fluctuation in 30-day mortality **(A)** and 1-year mortality **(B)**. Adjusted hazard ratio and 95% CI for HR fluctuation in 30-day mortality **(C)** and 1-year mortality **(D)**. The analyses used a model with restricted cubic splines. The reference (hazard ratio = 1, horizontal dotted line) was an HR fluctuation of 30 bpm (vertical dotted line). Adjusted variables included age, male sex, weight, SOFA score, admission HR, sedative use, ventilation use, vasopressor use, CHF, AF, hypertension, and liver disease, namely, model 5 described above. HR, heart rate; SOFA, sequential organ failure assessment; CHF, congestive heart failure; AF, atrial fibrillation; CI, confidence interval.

To better investigate the potential non-linear association between HR fluctuation and mortality, the relative hazard compared with an HR fluctuation of 30 bpm as a reference was calculated. The association between HR fluctuation and mortality was similar in crude and adjusted models, with an increased risk in patients with HR fluctuation > 30 bpm. There was a typical J-type curve observed in restricted cubic splines for the association between HR fluctuation and 30-day or 1-year mortality of MI patients, with the lowest risk on the HR fluctuation of 30 bpm (Figure 1).

**Figure 2:**
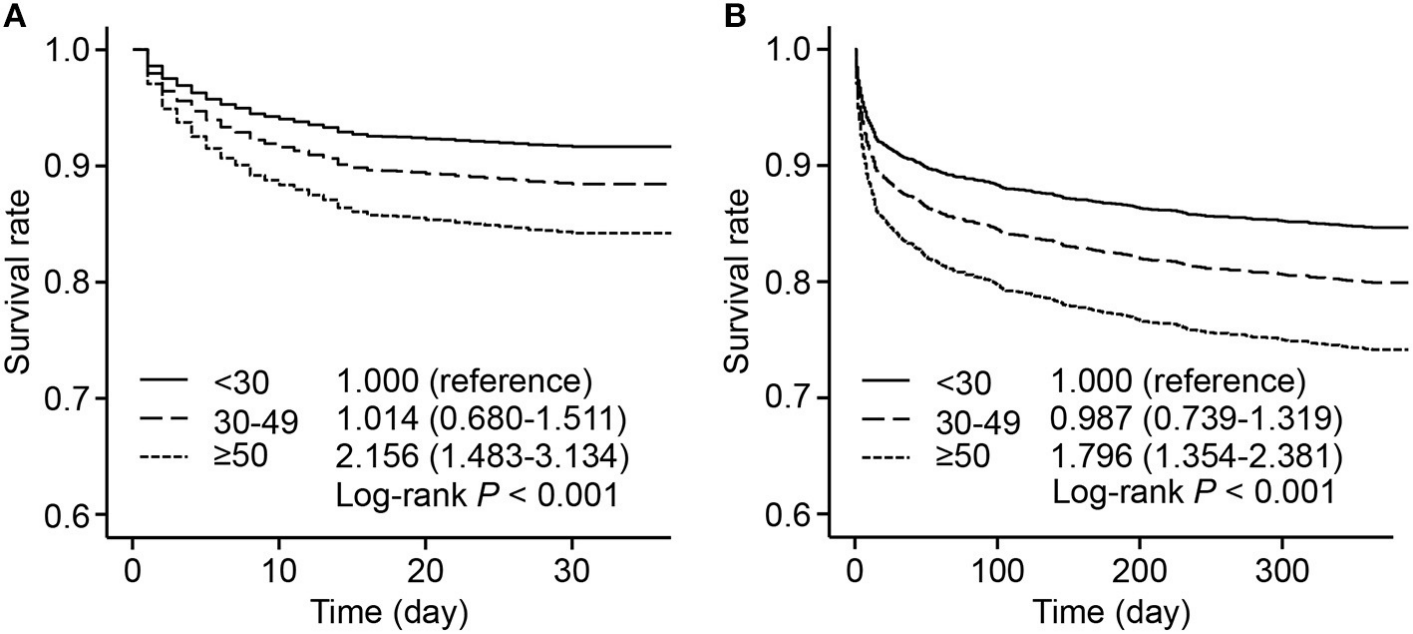
Adjusted survival rate by 3 HR fluctuation groups. Survive curves presented adjusted survival rates of 30-day mortality **(A)** and 1-year mortality **(B)** by 3 HR fluctuation groups. Adjusted variables included age, male sex, weight, SOFA score, admission HR, sedative use, ventilation use, vasopressor use, CHF, AF, hypertension, and liver disease, namely, model 5 described above. The hazard ratio and 95% CI were calculated compared with those of the low HR fluctuation group. HR, heart rate; SOFA, sequential organ failure assessment; CHF, congestive heart failure; AF, atrial fibrillation; CI, confidence interval.

The survival rates were presented by 3 groups with different levels of HR fluctuation. Compared with the low HR fluctuation group, the high HR fluctuation group suffered a significantly higher risk of mortality after adjustment, with hazard ratios of 2.156 (95% CI, 1.483–3.134) for 30-day mortality and 1.796 (95% CI, 1.354–2.381) for 1-year mortality (Figure 2).

**Figure 3:**
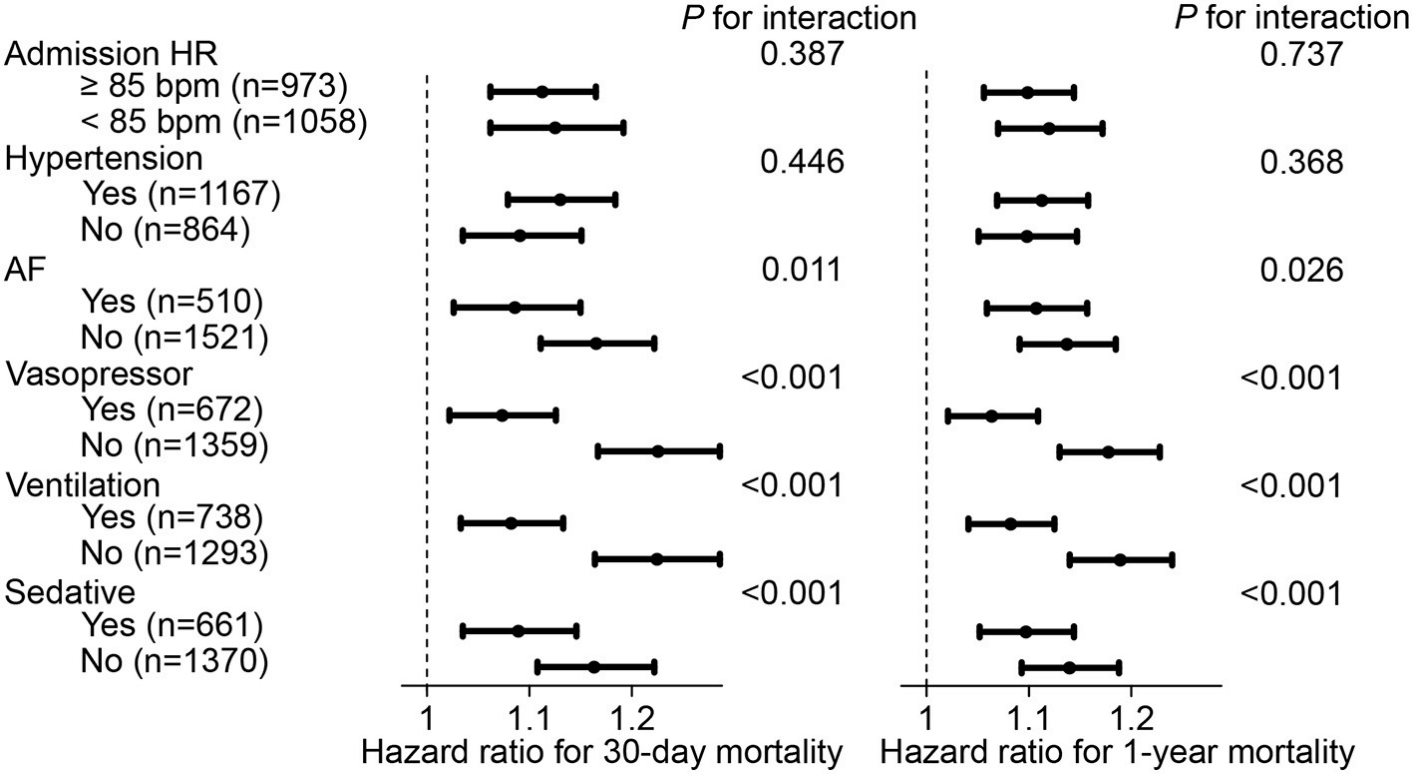
Hazard ratio and 95% CI of HR fluctuation (continuous) for mortality in subgroups. Hazard ratio and 95% CI were calculated along as 10 bpm HR fluctuation increase by Cox hazard regression model using backward process. Model adjusted for age, male, weight, SOFA score, admission HR, sedative, ventilation, vasopressor, CHF, AF, hypertension, liver disease, namely model 5 described above. HR, heart rate; SOFA, sequential organ failure assessment; CHF, congestive heart failure; AF, atrial fibrillation; CI, confidence interval.

Subsequently, to determine the influence of admission HR on our results, all participants were divided into 2 subgroups with a cut-off value of admission HR at 85 bpm for further sensitivity analysis. In the subgroup with admission HR ≥ 85 bpm, each 10-bpm increase in HR fluctuation was correlated with an increased risk of 30-day mortality (hazard ratio, 1.112, 95% CI, 1.062–1.165) and 1-year mortality (hazard ratio, 1.099, 95% CI, 1.056–1.144). This result was also observed in participants who had an admission HR <85 bpm. Furthermore, sensitivity analyses were also conducted for comorbidities of hypertension, AF, vasopressor use, ventilation use, and sedative use, which showed that the positive correlation between HR fluctuation and the risk of mortality was still statistically significant (each *P* < 0.05) (Figure 3).

## Discussion

In this retrospective cohort study with critically ill MI patients in the ICU, we found that the HR fluctuation was significantly associated with 30-day mortality and 1-year mortality, even after a broad array of potential confounders were taken into account. This association was independent of admission resting HR, comorbidities of hypertension or AF, or the use of ventilation or sedatives. Furthermore, a typical J-type curve was observed in restricted cubic splines for the association between HR fluctuation and 30-day or 1-year mortality of MI patients, indicating the lowest hazard on the HR fluctuation of 30 bpm. To the best of our knowledge, this is the first study clearly revealing the association between HR fluctuation and the risk of mortality in MI patients and highlighting that HR fluctuation may be a useful and easily measured marker as well as a prognostic predictor in clinical practice.

HR has been shown to be associated with cardiovascular outcomes in various large cohorts (11, 12). Additionally, beat-to-beat variations in HR have been reported to offer additional prognostic information for chronic heart failure, probably by reflecting autonomic dysfunction (13, 14). However, heart rate variability was complexly measured depending on ambulatory electrocardiography and limited to patients with sustained periods of normal sinus rhythm (15). HR fluctuation, calculated as the maximum minus the minimum HR using the initial 24-h data, showed its strength as a simpler and more generalizable marker. Recently, HR fluctuation was demonstrated to predict mortality and hospitalization in chronic heart failure (6). Another study showed that both high and low HR fluctuation were harmful for survival of critically ill patients in ICU (7). However, this current study focused on MI patients and found that HR fluctuation was positively associated with 30-day mortality and 1-year mortality in MI patients, emphasizing its feasibility and importance for outcome prediction of MI.

Indeed, HR may be easily affected by the use of drugs or other therapies, such as antihypertensives, antiarrhythmics, vasopressors, ventilation, and sedatives. In a large cohort with 15,680 community participants, β-blocker use at any time during the study could alter the association between HR change from the preceding visit and outcomes, leading to an association that was no longer significant between change in HR and all-cause mortality and incident HF (16). In another cohort of hypertensive patients, HR remained a significant independent risk factor for cardiovascular mortality after correction for HR-limiting therapy (17). In our study, history of hypertension, AF, vasopressor use, ventilation use, and sedative use were included in the sensitivity analysis, showing the consistent correlation between HR fluctuation and the mortality of MI patients in the majority subgroups. Because admission HR was reported to be associated with the severity and complexity of coronary artery disease, a sensitivity analysis was also conducted to determine the potential influence of different admission HRs (18). Surprisingly, our results remained significant in subgroups with different levels of admission HR, demonstrating that HR fluctuation is a reliable risk factor for mortality in MI patients.

It should be noted that the risk correlated with an HR increase is not always strictly fitted to a linear association because it is evident that both too high or too low of an HR could be harmful. A non-linear curve was observed for a trend between the mean HR and incident stroke in diabetes patients, which indicated the lowest risk for the mean HR of 65 bpm (11). However, an increased risk of all-cause mortality, along with an increase in HR changes from the preceding visit, was reported in a community population (16). Our restricted cubic splines clearly showed a J-type curve for the association between HR fluctuation and the 30-day or 1-year mortality of MI patients. According to the restricted cubic spline plots, patients with HR fluctuation of 10 or 20 bpm were with higher risk but this association did not achieve statistical significance. Interestingly, for both the 30-day and 1-year mortality of MI patients, the lowest risk of HR fluctuation was ~30 bpm, which might be a candidate marker for decision making in HR control strategies.

This study has some limitations. Our study was based on real-world clinical data, in which different intervals between HR monitoring measurements for each patient may exist. We calculated the HR fluctuation using the maximum and minimum HR across the initial 24 h and further adjusted for multiple covariates in the regression models to diminish their potential influence on mortality. Another limitation is that selection bias is inevitable in a retrospective study design, and future randomized controlled trials would help validate our findings.

## Conclusion

In conclusion, this retrospective cohort study revealed a positive association between HR fluctuation and 30-day and 1-year mortality in critically ill MI patients in the ICU. This association was not affected by the comorbidities of hypertension and AF or by the use of ventilation and sedatives. HR fluctuation might be an accessible and reliable marker and risk factor for mortality in critically ill patients, which is worthy of further investigation.

## Data Availability Statement

Publicly available datasets were analyzed in this study. This data can be found here: https://physionet.org/content/mimiciii/1.4/.

## Ethics Statement

Ethical review and approval was not required for the study on human participants in accordance with the local legislation and institutional requirements. Written informed consent for participation was not required for this study in accordance with the national legislation and the institutional requirements.

## Author Contributions

QG, JiW, and YZ conceived and designed the research protocol. QG, HL, and RS collected and analyzed the data, and wrote the first draft of the manuscript. All authors provided input on data analysis and interpretations, and participated in multiple revisions of the manuscript and approved the final version of the manuscript and agree to be accountable for all aspects of the work.

## Conflict of Interest

The authors declare that the research was conducted in the absence of any commercial or financial relationships that could be construed as a potential conflict of interest.
